# Nudging transparent behavioural science and policy

**DOI:** 10.1017/bpp.2018.10

**Published:** 2018-11

**Authors:** OLIVIA M. MAYNARD, MARCUS R. MUNAFÒ

**Affiliations:** 1 MRC Integrative Epidemiology Unit, University of Bristol, Bristol, UK and UK Centre for Tobacco and Alcohol Studies, School of Experimental Psychology, University of Bristol, Bristol, UK; 2 MRC Integrative Epidemiology Unit, University of Bristol, Bristol, UK and UK Centre for Tobacco and Alcohol Studies, School of Experimental Psychology, University of Bristol, Bristol, UK

## Abstract

There are inherent differences in the priorities of academics and policy-makers. These pose unique challenges for teams such as the Behavioural Insights Team (BIT), which has positioned itself as an organisation conducting academically rigorous behavioural science research in policy settings. Here we outline the threats to research transparency and reproducibility that stem from working with policy-makers and other non-academic stakeholders. These threats affect how we perform, communicate, verify and evaluate research. Solutions that increase research transparency include pre-registering study protocols, making data open and publishing summaries of results. We suggest an incentive structure (a simple ‘nudge’) that rewards BIT's non-academic partners for engaging in these practices.

Since its inception in 2010, the Behavioural Insights Team (BIT) has positioned itself as an organisation conducting academically rigorous behavioural science research in policy settings. Over the past six years they have conducted over 300 randomised controlled trials (RCTs), with some of these published in peer-reviewed journals. Their findings have been used to inform policy around the world and changed the way governments, businesses and other organisations operate. Their recent collaborators and funders include the UK government (e.g., Cabinet Office, Department of Business Innovation and Skills), overseas governmental organisations (e.g., the Guatemalan tax authority) and non-profit organisations including charities and the World Bank.

While working with these organisations provides unique opportunities, it also poses unique challenges for a team conducting academic research. Not only must the research meet the needs of these partner organisations, but it must be of the highest quality if it is to contribute to current scientific thinking.

There has been considerable debate in recent years over the reproducibility and transparency of scientific research (Ioannidis, 2005). In their review of the work of BIT published in this edition of *Behavioural Public Policy*, Sanders and colleagues (2018) discuss what they refer to as the reproducibility crisis: “The crisis should not be dismissed as of merely academic interest, since several of these findings are ones that have been – or could be – applied to policy problems.”

Acknowledging the problem is one thing – accepting that *all* those who conduct research are part of the solution is another. The field of meta-science (Ioannidis *et al.*, 2015) is rapidly developing strategies to improve the transparency and robustness of research (Ioannidis, 2014; Munafò *et al.*, 2017). These include improving the research process at all stages, including how we perform, communicate, verify and evaluate research (Ioannidis *et al.*, 2015). However, as we discuss here, conducting research in settings so closely linked with policy poses unique challenges, including time pressures, short decision-making cycles, conscious and unconscious biases, vested interests and lack of incentives to conduct academically rigorous research. We outline the threats to reproducibility that stem from working with policy-makers and other non-academic stakeholders and provide a starting point for developing solutions to these challenges.

## Transparent performance of research

There are inherent differences in the priorities of academics and policy-makers. Academics are incentivised to generate research that is publishable, leads to funding and, to some extent, is translatable. In comparison, governments and other authorities are interested in the profitability and translatability of research (Ioannidis, 2014). These differences in priorities mean that the research conducted by teams such as BIT in collaboration with policy-makers is typically much more rapidly translated and applied to policy settings than research conducted in traditional academic settings. However, as Sanders and colleagues (2018) describe, this has meant that (until recently) their research efforts have been focused on the “low-hanging fruit”; projects that are more likely to be of interest to policy-makers, but perhaps of less scientific interest.

Sanders and colleagues (2018) also discuss how a misalignment of research priorities means that “there may be differences over the proposed timing, framing and conclusions of any potential publication.” While academics may engage in HARKing (Hypothesising After the Results are Known) in order to generate publishable results (Kerr, 1998), policy-makers or private investors may be motivated to ensure that research findings are in line with their interests, and may therefore be prone to CoRKing (our own phrase – ‘Concluding before the Results are Known’).

Pre-registration of study protocols on platforms such as the Open Science Framework (Foster & Deardorff, 2017) or the ISRCTN registry (https://www.isrctn.com) creates a permanent record of the protocol prior to the start of testing. By including a data analysis plan and a comprehensive record of all outcome measures, pre-registration makes it harder for those with vested interests from consciously or unconsciously HARKing or CoRKing (Rifai *et al.*, 2014; Munafò *et al.*, 2017) and increases the methods reproducibility of the research (see Box 1). BIT have already started to engage in pre-registration practices, although there is room for improvement: at the time of our searches (in October 2017), of the 11 studies published by BIT in academic journals, only one (Hallsworth *et al.*, 2016) has an accompanying study protocol on the BIT website (Chadborn & Sanders, 2014). Protocols can be pre-registered but embargoed for a period (the Open Science Framework has this functionality) where those commissioning the research are concerned about making research plans public (especially where the research involves changing the behaviour of the general public). Once the research is complete, the protocol can be made public and will retain its date stamp proving its pre-registration status.

Box 1. Defining reproducibility.

Goodman and colleagues (2016) provide a conceptual framework for terms related to ‘research reproducibility’, which is summarised below.

*Methods reproducibility* **–** are the methods provided in enough detail such that they can be implemented and exactly repeated?

*Results reproducibility (also ‘replicability’)* – can the results be duplicated if the same procedures are followed with new data?

*Inferential reproducibility* **–** are qualitatively similar conclusions drawn from independent replications or re-analysis of the original dataset?

*Robustness and generalisability* **–** do the results remain the same in settings different from the original experimental framework?

## Transparent verification of research

Sanders and colleagues (2018) describe how senior officials are particularly concerned about “any transfer of data … (even anonymised data).” This concern is not unique to policy-makers, as academics have also been shown to be reluctant to share their data (Wicherts *et al.*, 2006). However, the benefits of open data (and accompanying meta-data) are compelling. Open data means that results can be reproduced independently and verified, interventions can be better understood, alternative explanations can be explored and CoRKing can be identified. Together, this is a way of assessing the inferential reproducibility of research (see Box 1). Recent initiatives spearheaded by individual researchers (Nosek *et al.*, 2015; Morey *et al.*, 2016), publishers and research councils have encouraged the sharing of study data. Study data can be published on the Open Science Framework alongside pre-registered study protocols.

## Transparent communication of research

There is evidence of publication bias in BIT. Sanders and colleagues (2018) distinguish between “initiatives that are not made public and those that were made public but did not go through the additional step of peer-reviewed publication.”

As studies are typically not pre-registered, the extent of any file drawer of unpublished studies (i.e., publication bias) in BIT is unknown. However, with over 300 RCTs conducted over the past six years, the 30 ‘academic publications’ on their website and 39 ‘policy publications’ at the time of our searches (in October 2017) are unlikely to tell the whole story of BIT activity. As Sanders and colleagues (2018) acknowledge, publication bias reduces the “transparency of government,” causing a “‘public file drawer’ problem” that distorts the literature, ultimately to the detriment of future research and policy practices.

The reasons for publication bias have been described elsewhere (Rosenthal, 1979; Joober *et al.*, 2012; Franco *et al.*, 2014; Ioannidis *et al.*, 2014). However, they are worth describing here in the context of research conducted in policy settings. An underlying reason for publication bias in both academic and policy settings is the pressure to ‘find’ interesting results or perhaps, in the case of policy-makers, findings that fit with their policy objectives. It is also widely acknowledged that null findings are often not made public (Franco *et al.*, 2014). Withholding null findings can have serious implications for the transparency of research. Rather than being seen as interventions that failed to ‘work’, null findings resulting from well-designed and adequately powered studies should be made public in order to prevent the draining of valuable resources into research questions that have already been answered. Increasingly, there is recognition of the importance of reporting null findings, and it is encouraging that BIT have recently published null findings from a trial investigating methods of priming honesty among individuals completing tax returns (Kettle *et al.*, 2017).

Another underlying reason for publication bias is a lack of time to devote to writing up ‘uninteresting’ findings. As Sanders and colleagues (2018) say, “there are often few resources provided to support the publication process, which is likely to be seen as a luxury.” Time is not a luxury many policy-makers have. Importantly, for teams such as BIT, their findings can alter government action as soon as the results are in, long before publication in an academic journal. Where the impact comes first, and those commissioning the work have already moved to the next problem, publication in an academic journal becomes less of a priority. Where time and limited resources would otherwise prevent publication of manuscripts, BIT could consider working with academic partners, for whom publishing in academic journals is not a luxury, but a necessity. BIT has a history of employing PhD students to conduct research internships and, using this scheme, a mutually beneficial relationship could be established such that the student gains experience working in a policy setting while writing up a manuscript for publication that otherwise would not have been published. As a minimum, brief summaries of research findings could be published online (on the Open Science Framework, for example) alongside the pre-registered protocol and data. In this instance, this final step would be trivial, but would ensure that research findings are made open. Together, these components could be ‘published’ by assigning a DOI to the deposit, which would allow other researchers to cite it.

## Transparent evaluation of research

Where BIT findings are written up, these are rarely published in peer-reviewed journals. Of the 30 academic publications on their website, 11 have been published in academic journals and thus peer reviewed. Where publication in academic journals is not possible, Sanders and colleagues (2018) suggest that manuscripts should “provide enough detail to allow a reasonable judge of their quality.” This is already encouraged for RCTs – the CONSORT statement (Schulz *et al.*, 2010) provides an evidence-based minimum set of requirements for reporting the results of RCTs. It also includes extensions for specific types of RCTs, such as cluster-randomised trials, which are often used by BIT (Campbell *et al.*, 2012). Using internationally recognised standards would increase the reproducibility of their research.

In recent years, peer review has been heavily criticised (Bohannon, 2013) and post-publication peer review has been provided as an alternative (Hunter, 2012; Kriegeskorte, 2012; Teixeira da Silva & Dobránszki, 2015). Using platforms such as PubPeer, academics can support independent scrutiny and comment on the research methodology, the reliability of the findings and the appropriateness of the conclusions. Arguably, this kind of evaluation is particularly important for the research conducted by BIT, given that many of the users of their research are not academics but policy-makers and members of similar teams worldwide who may have less experience in evaluating research designs and findings.

## A nudge towards transparency

We suggest an incentive structure that rewards BIT's non-academic partners for engaging in practices that increase research transparency. Munafò and colleagues (2017) describe how providing incentives can increase the uptake of practices promoting open science. For example, badges for open science practices that accompany published manuscripts may increase the uptake of these practices (Kidwell *et al.*, 2016). No doubt BIT would consider these a simple, low-cost and effective ‘nudge’, increasing the transparency and reproducibility of the research reported.

**Figure 1.**
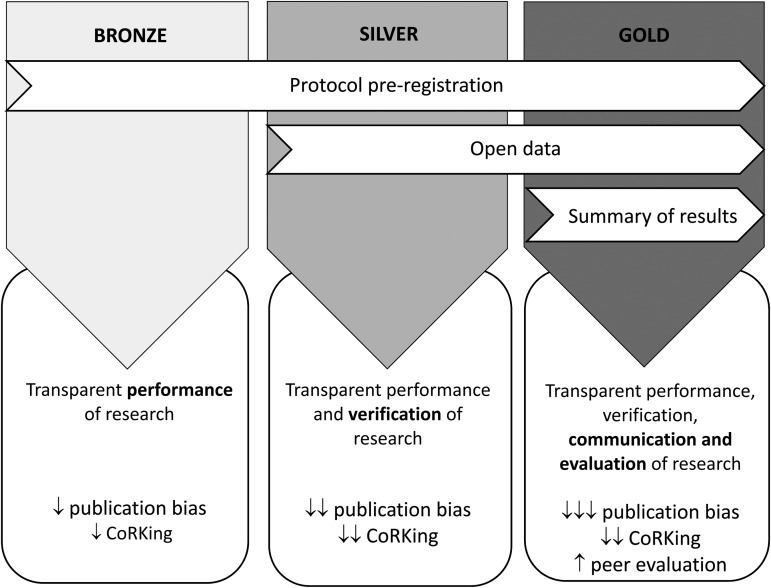
Tiered incentive structure to encourage transparent performance, verification, communication and evaluation of research. CoRKing = Concluding before the Results are Known.

We envisage a tiered approach (see Figure 1) where those commissioning research can choose from the bronze, silver and gold options. Research can then be badged on the BIT website under one of these headings. This has the additional benefit of bringing the focus onto those commissioning the research, rather than BIT themselves. This is important given that BIT have been taking important steps to increase the transparency of their research, with these steps arguably hampered by those they work with.

The bronze level requires that a study protocol is registered online prior to the start of testing. The silver level requires data (and accompanying meta-data) to also be published, and the gold level requires a summary of the data to be published once the data have been analysed. Where time and resources permit, a pre-print or working paper that follows reporting guidelines (such as CONSORT; Schulz *et al.*, 2010) could be published, or a manuscript could be submitted to a peer-reviewed journal. Protocols, data and results could all be published in a single location, such as the Open Science Framework, and assigned a persistent link via a DOI.

## Conclusions

For those such as BIT, who are conducting research at the interface between academia and policy-making, there are unique challenges including time pressures, conflicts of interest and biases. These impact how research is conducted and reported, including which conclusions are drawn, which findings are made public and which findings are published in academic journals.

We have only examined how pressures from stakeholders and policy-makers can influence certain aspects of how research is performed, reported, verified and evaluated. However, there are other elements of the research process, including the development of research questions and the analysis of data, which may face unique challenges in these settings. After careful consideration of how research such as that conducted by BIT may be affected in these areas, we expect that our incentive framework could be developed to incorporate methods of reducing these additional threats to reproducibility and transparency.

Finally, it is important to acknowledge that the work being conducted by BIT is unprecedented in terms of its scope, reach and the degree to which is has already contributed to scientific knowledge. For an organisation not officially affiliated with a university, its publication record is impressive. Our review aims to provide some concrete examples of how this track record can be further enhanced and how BIT can serve as an example to the other teams using ‘behavioural insights’ globally. As Sanders and colleagues (2018) say, “what the global community of behavioural scientists does next will determine whether policy-makers will continue to see behavioural science as a reliable source of policy ideas and approaches.”
